# MRI and active surveillance: thoughts from across the pond

**DOI:** 10.1007/s00330-024-10866-6

**Published:** 2024-09-12

**Authors:** Rebecca A. Campbell, Andrew Wood, Zeyad Schwen, Ryan Ward, Christopher Weight, Andrei S. Purysko

**Affiliations:** 1 Glickman Urological and Kidney Institute, Cleveland Clinic, Cleveland, OH USA; 2 Abdominal Imaging Section, Diagnostics Institute, Cleveland, OH USA

**Keywords:** Prostate cancer, Watchful waiting, Multiparametric magnetic resonance imaging

## Abstract

**Abstract:**

In the United States (US), urological guidelines recommend active surveillance (AS) for patients with low-risk prostate cancer (PCa) and endorse it as an option for those with favorable intermediate-risk PCa with a > 10-year life expectancy. Multiparametric magnetic resonance imaging (mpMRI) is being increasingly used in the screening, monitoring, and staging of PCa and involves the combination of T2-weighted, diffusion-weighted, and dynamic contrast-enhanced T1-weighted imaging.

The American Urological Association (AUA) guidelines provide recommendations about the use of mpMRI in the confirmatory setting for AS patients but do not discuss the timing of follow-up mpMRI in AS. The National Comprehensive Cancer Network (NCCN) discourages using it more frequently than every 12 months. Finally, guidelines state that mpMRI can be used to augment risk stratification but should not replace periodic surveillance biopsy.

In this review, we discuss the current literature regarding the use of mpMRI for patients with AS, with a particular focus on the approach in the US. Although AS shows a benefit to the addition of mpMRI to diagnostic, confirmatory, and follow-up biopsy, there is no strong evidence to suggest that mpMRI can safely replace biopsy for most patients and thus it must be incorporated into a multimodal approach.

**Clinical relevance statement:**

According to the US guidelines, regular follow-ups are important for men with prostate cancer on active surveillance, and prostate MRI is a valuable tool that should be utilized, in combination with PSA kinetics and biopsies, for monitoring prostate cancer.

**Key Points:**

- *According to the US guidelines, the addition of MRI improves the detection of clinically significant prostate cancer.*
- *Timing interval imaging of patients on active surveillance remains unclear and has not been specifically addressed.*
- *MRI should trigger further work-ups, but not replace periodic follow-up biopsies, in men on active surveillance.*

## Introduction

Active surveillance (AS) for prostate cancer (PCa) encompasses various protocols aimed at avoiding or delaying curative treatment for men with favorable-risk cancers and life expectancy greater than 10 years and thus avoiding the side effects that are inherent to all PCa treatments [1, 2]. Programs include three components: (1) selection criteria (D’Amico or National Comprehensive Cancer Network (NCCN) risk categories); (2) monitoring strategy (i.e., frequency of prostate-specific antigen (PSA) levels, digital rectal exam, and confirmatory prostate biopsy), and (3) triggers for intervention (often upgrading to ≥ Grade Group (GG) 2).

Studies support the use of AS, showing that many patients can avoid treatment while maintaining excellent cancer-specific survival [3, 4]. Numerous large studies show increasing use of AS over time in the United States (US), although it remains under-utilized [5–8].

Most programs include confirmatory biopsy within approximately 1 year of diagnosis, with the goal of facilitating early detection of those who were under-sampled initially or identifying those at higher risk of future reclassification [9–13].

Despite national guidelines’ recommendations for confirmatory biopsy, US studies have shown that it is not uniformly performed in real-world practice, likely due to patient discomfort, cost, and complications [9]. Thus, there remains a need for non-invasive and cost-effective tests for patients on AS to detect progression to clinically significant PCa (csPCa).

One non-invasive tool for both diagnosis and monitoring is multiparametric magnetic resonance imaging (mpMRI), which consists of a combination of T2-weighted, diffusion-weighted, and dynamic contrast-enhanced T1-weighted imaging. The Prostate Imaging Reporting and Data System (PI-RADS) is the standardized scoring system used to predict the likelihood of csPCa based on mpMRI findings [10].

Current literature regarding the use of mpMRI for patients on AS shows a benefit to the addition of mpMRI to diagnostic, confirmatory, and follow-up biopsy. However, there is no strong evidence to suggest that mpMRI can safely replace biopsy for most patients and thus should be used within a multimodal approach. Furthermore, current data is lacking about the ideal imaging interval for patients on AS.

In this review, we will discuss the current guidelines and literature regarding the use of mpMRI of the prostate during AS, with a particular focus on the approach in the US.

### US guidelines and comparison to European guidelines

The American Urological Association (AUA) guidelines recommend AS as the preferred option for patients with very low and low-risk PCa and NCCN guidelines recommend AS for most patients with very low or low-risk PCa. They also advise that AS be offered to patients with favorable intermediate-risk PCa, along with radiation therapy and radical prostatectomy. Furthermore, AUA guidelines recommend a patient undergo mpMRI if the diagnostic biopsy was performed without mpMRI guidance, followed by a timely confirmatory targeted biopsy if a PI-RADS 4–5 lesion is present. If no suspicion for csPCa is present on mpMRI, then a confirmatory biopsy can be performed within 12 months of diagnosis [11, 12].

US guidelines do not recommend a specific imaging interval for patients on AS, however, the NCCN discourages the use of mpMRI more frequently than every 12 months [13]. The AUA guidelines specifically state that mpMRI can be used to augment risk stratification but should not replace periodic surveillance biopsy.

The European guidelines differ only slightly from the US. Similar to the AUA guidelines in regards to confirmatory biopsy as described above, the European Association of Urology (EAU) guidelines state that for men with a diagnostic biopsy that was systematic only, a confirmatory biopsy should be performed within 6–12 months and that this should be a combined systematic- and MRI-targeted biopsy [14, 15]. Furthermore, they cite the DETECTIVE study, which supports the omission of confirmatory biopsy in patients who were already deemed eligible for AS based on a combined biopsy [16]. This is similar to NCCN guidelines which state that early confirmatory testing may not be necessary for patients who had an mpMRI prior to diagnostic biopsy [13].

EAU guidelines specifically state that radiological progression on interval MRI should trigger further work-up with a biopsy but not active treatment. Addressing this topic slightly differently, AUA guidelines advise that mpMRI should not replace surveillance biopsy.

Overall, neither European nor US guidelines provide specific recommendations about the timing of mpMRIs for men on AS (Table 1). Differences between other aspects of the guidelines are likely explained by the varying times that each set of guidelines was formulated and released, as well as different approaches to the same topic.

**Table 1:** Comparison of US and European guidelines for the use of multiparametric prostate MRI in diagnosis of prostate cancer and active surveillance

Guidelines	Diagnostic setting	Repeat biopsy setting in those with prior negative biopsy	Confirmatory setting in AS	Follow-up/surveillance setting in AS
American Urologic Association (AUA) [9, 10, 50, 51]	• *May* use MRI prior to initial biopsy to increase detection of csPCa (Conditional Recommendation; Evidence Level: Grade B) • Radiologists *should* use PI-RADS in reporting MRI results (Moderate Recommendation; Evidence Level: Grade C). • If csPCa on MRI → clinicians *should* perform targeted biopsy ± systematic biopsy (Moderate Recommendation (targeted biopsies)/Conditional Recommendation (systematic template biopsy); Evidence Level: Grade C. • If no csPCa on MRI but the elevated risk for csPCa → clinicians *should* perform a systematic biopsy (Moderate Recommendation; Evidence Level: Grade C)	• If a prior negative biopsy was performed without MRI → clinicians *should* use MRI prior to repeat biopsy (Strong Recommendation; Evidence Level: Grade C). • If csPCa on MRI and undergoing repeat biopsy → clinicians *should* perform targeted biopsy ± systematic biopsy (Moderate Recommendation (targeted biopsies)/Conditional Recommendation (systematic template biopsy); Evidence Level: Grade C). • If no csPCa on MRI but indications for repeat biopsy → clinicians *may* perform a systematic biopsy (Conditional Recommendation; Evidence Level: Grade B)	• mpMRI may be used for risk stratification but should not replace repeat biopsies (Expert Opinion). • Obtain mpMRI if diagnostic biopsy was performed without mpMRI guidance. If PI-RADS 4–5 → timely confirmatory biopsy (targeted) should be performed. If PI-RADS 1–3 → confirmatory biopsy may be performed within approximately 12 months of diagnosis.^a^	• mpMRI may be used for risk stratification but should not replace repeat biopsies (Expert Opinion)
National Comprehensive Cancer Network (NCCN) [11, 52]	• Obtain mpMRI prior to biopsy if available (category 1)		• MRI with the calculation of PSA density and repeat biopsy as indicated is an option for confirmatory testing (within first 6–12 months) (category 2A). • Early confirmatory testing (within first 6–12 months) may not be necessary for those who had mpMRI prior to diagnostic biopsy (category 2A)	• Surveillance MRI should not be performed more often than every 12 months unless clinically indicated (category 2A)
European Association of Urology (EAU) [12, 15]	• Perform MRI before prostate biopsy (Strong). • For biopsy naïve: If PI-RADS ≥ 3 → combine targeted and systematic biopsy (Strong). If negative MRI and low suspicion for csPCa → omit biopsy based on shared decision-making (Weak).	• Perform MRI before prostate biopsy (Strong). • For prior negative biopsy: If PI-RADS ≥ 3 → perform targeted biopsy only (Weak). If negative MRI and high suspicion for csPCa → perform a systematic biopsy based on shared decision-making (Strong).	• Obtain MRI before confirmatory biopsy if no MRI prior to diagnostic biopsy (Strong). • Obtain both targeted biopsy (for any PI-RADS ≥ 3) and systematic biopsy if a confirmatory biopsy is performed (Strong). • If the patient had an MRI with an initial biopsy, and both targeted and systematic biopsies were performed, then confirmatory biopsy can be omitted (Weak).	• Perform MRI and repeat biopsy if PSA doubling time < 3 years (Strong). • Intervention should be triggered by changes on biopsy, not on progression on MRI (Weak). • Patients with PI-RADS 1–2 on MRI and PSA density < 0.15 may be exempt from repeat biopsy (Weak)

*AS* active surveillance, *AUA* American Urological Association, *csPCa* clinically significant prostate cancer (Grade Group 2 or higher), *EAU* European Association of Urology, *MRI* magnetic resonance imaging, *PI-RADS* Prostate Imaging Reporting and Data System, *PSA* prostate-specific antigen

AUA recommendation levels: Evidence level: A = very confident; B = moderately confident; C = limited confidence or very little confidence. Evidence grade: strong recommendation = Net benefit or harm substantial; Moderate Recommendation = Net benefit or harm moderate; Conditional Recommendation = Net benefit or harm comparable to other options; Clinical Principle = Widely agreed upon by urologists or other clinicians for which there may or may not be evidence; Expert Opinion = Consensus of the Panel, based on members’ clinical training, experience, knowledge and judgment for which there may or may not be evidence

NCCN recommendation levels: Category 1 = Based upon high-level evidence, there is uniform NCCN consensus that the intervention is appropriate; Category 2A = Based upon lower-level evidence, there is uniform NCCN consensus that the intervention is appropriate; Category 2B = Based upon lower-level evidence, there is NCCN consensus that the intervention is appropriate; Category 3 = Based upon any level of evidence, there is major NCCN disagreement that the intervention is appropriate

EAU recommendation levels: “Strong” or “Weak” based on GRADE methodology

^a^This recommendation is included within the text discussion of Guidelines Statement 18 (mpMRI may be used for risk stratification but should not replace repeat biopsies) which was rated Expert Opinion

### Role, advantages, and limitations of MRI

#### Baseline/diagnostic and early re-biopsy setting

**Fig. 1:**
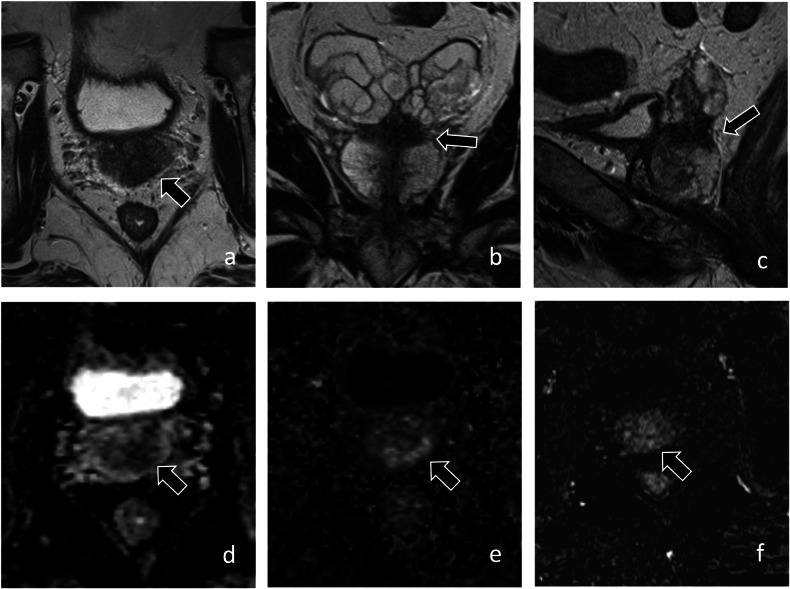
68-year-old man with PSA 6.5 ng/mL and grade group 1 prostate cancer detected on systematic biopsy. MRI was performed prior to confirmatory biopsy. Axial (**a**), coronal (**b**), and sagittal (**c**) T2-weighted images show a PI-RADS 5 lesion (arrow) involving the peripheral and central zone of the prostate with the invasion of the seminal vesicles. The lesion demonstrates markedly low signal intensity on the apparent diffusion coefficient map (arrow, **d**), markedly high signal on high *b*-value (1400 s/mm^2^) diffusion-weighted images (arrow, **e**), and early enhancement on T1-weighted dynamic contrast-enhanced images (arrow, **f**). MRI-guided biopsy showed grade group 5 cancer with extra-prostatic extension. The patient underwent radical prostatectomy, which revealed grade group 5 cancer, pT3b N1

Baseline/initial biopsy performed with combined MRI-targeted and systematic cores has been shown to reduce the risk of misclassification compared to systematic biopsy alone. One study compared men with initial systematic biopsies to men with MRI-targeted biopsies, all of whom underwent combined follow-up biopsies. Of those initially diagnosed with systematic biopsy alone, 59% were pathologically upgraded at follow-up compared to 19% in the cohort that underwent initial MRI-targeted biopsy [17]. Another study supported the early use of mpMRI following initial biopsy. The ROMAS trial randomized patients who were initially diagnosed with systematic biopsy to a study group that underwent mpMRI at 3 months from AS enrollment compared to a control group that did not undergo mpMRI. Positive mpMRI was observed in 34% of patients in the study group and 12% of these patients were reclassified at 3-month MRI-targeted biopsy. All patients underwent systematic biopsy at 12 months, at which time reclassification was 7% for the study group compared to 29% for the control group (*p* < 0.01) [18]. These results support the early use of mpMRI either at initial biopsy or early in AS if not previously obtained to reduce the risk of later reclassification (Fig. 1).

#### Confirmatory biopsy setting

Combined biopsy in the confirmatory biopsy setting for men on AS has been associated with fewer negative confirmatory biopsies and detection of more csPCa compared to systematic biopsy alone. The ASIST trial randomized 273 men on AS to systematic or mpMRI-targeted confirmatory biopsy which was to occur at 9–13 months following diagnosis. mpMRI-targeted confirmatory biopsy resulted in 50% fewer AS failures (19% versus 35%, *p* = 0.02) and less progression to csPCa (10% versus 23%, *p* = 0.048) at 2-year follow-up [19]. Shapiro et al matched patients on AS into two cohorts including those with systematic alone versus those with combined confirmatory biopsy which was completed within 24 months of diagnosis. Following confirmatory biopsy, 46% of patients in the combined biopsy cohort were pathologically upgraded compared to only 18% in the systematic biopsy cohort (*p* < 0.001). Furthermore, the rate of negative confirmatory biopsy was 13% in the combined biopsy cohort compared to 38% in the systematic biopsy cohort (*p* < 0.01). Confirmatory combined biopsy was significantly associated with pathologic upgrading compared to systematic biopsy alone (OR 3.6), showing that combined biopsy was better at detecting csPCa and excluding patients from AS [20].

Additionally, a systematic review and meta-analysis showed that MRI-targeted biopsies alone in the confirmatory biopsy setting (6–24 months after diagnosis) would have missed upgrading to csPCa in 10% of patients and systematic biopsies alone would have missed upgrading in 7% [21]. Current literature supports a combined biopsy approach for confirmatory biopsy [22].

A negative MRI-targeted confirmatory biopsy also yields important prognostic information including a favorable prognosis for the ability to remain on AS long-term. Bloom et al examined patients with GG1/GG2 PCa who underwent mpMRI-guided biopsy after referral (mean 12.2–16.4 months after referral). Median progression-free survival was longer in the negative mpMRI-guided biopsy cohort (74 months versus 44 months, *p* < 0.01) and 62% of patients in the positive MRI-targeted biopsy cohort remained on AS compared to 92% in the negative mpMRI-guided biopsy cohort (*p* < 0.01) [23]. Overall, these findings suggest that patients with a negative mpMRI-targeted confirmatory biopsy on AS have a favorable prognosis in terms of pathologic progression and ability to remain on AS and could be counseled that longer intervals between imaging and biopsy may be reasonable.

#### Follow-up biopsy setting

Studies also support the use of mpMRI with follow-up biopsies for patients on AS. A National Cancer Institute study compared those with systematic biopsy, mpMRI-targeted biopsy, and combined biopsy at 2 years and found that grade progression was detected in 16%, 26%, and 33%, respectively. At 2 years, mpMRI-targeted biopsy alone detected over 51% of patients who had grade progression and thus if only systematic biopsy had been performed, half of all patients with progression would have been missed. For patients followed for 4 years and 6+ years, mpMRI-targeted biopsy alone detected more pathologic progression compared to systematic biopsy alone at these time points, thus supporting the use of mpMRI-targeted biopsy at surveillance intervals [24].

#### Negative predictive value of baseline negative mpMRI

**Fig. 2:**
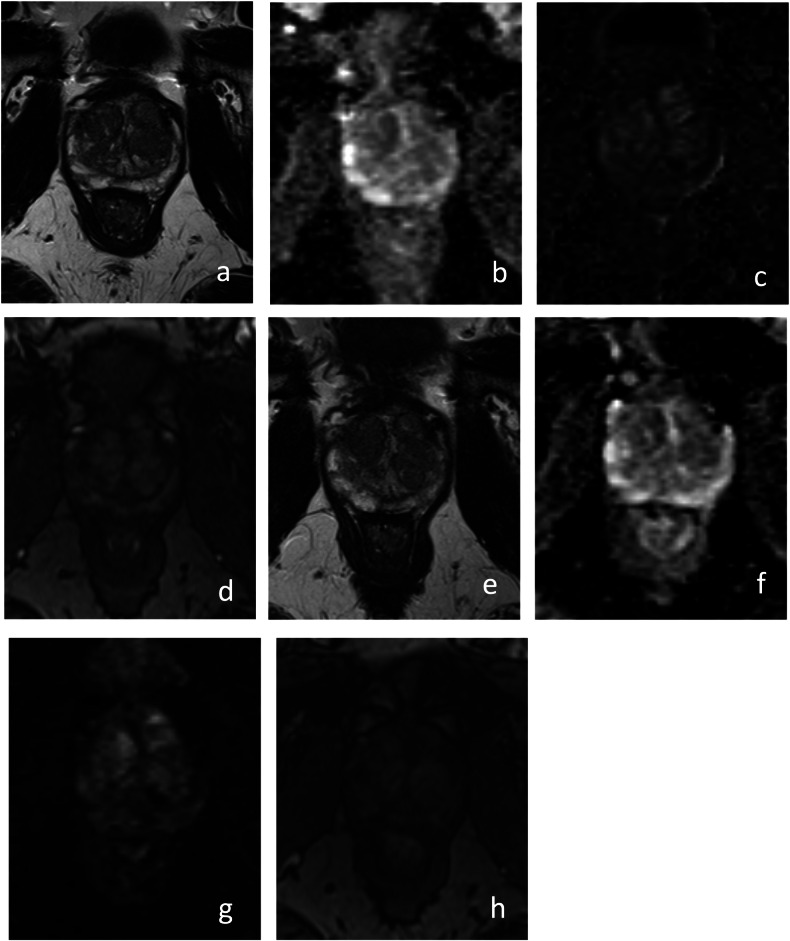
64-year-old man with PSA of 5.9 ng/mL and grade group 1 prostate cancer detected on systematic biopsy. Baseline multiparametric MRI performed prior to surveillance biopsy revealed no PI-RADS 3–5 lesions on axial T2-weighted (**a**), apparent diffusion coefficient map (**b**), high *b*-value (1400 s/mm^2^) diffusion-weighted (**c**), and T1-weighted dynamic contrast-enhanced images (**d**). Similarly, multiparametric MRI performed on subsequent follow-up revealed no PI-RADS 3–5 lesions on axial T2-weighted (**e**), apparent diffusion coefficient map (**f**), high *b*-value (1400 s/mm^2^) diffusion-weighted (**g**), and T1-weighted dynamic contrast-enhanced images (**h**) (PRECISE score: 3-NonV). Subsequent biopsy showed grade group 1 cancer and the patient has been on surveillance for 9 years

In general, studies have shown that negative mpMRI results for patients on AS are better at predicting biopsy results than positive mpMRIs. mpMRI is particularly effective at ruling out csPCa and has a strong negative predictive value (NPV) (Fig. 2). One study examined 300 men on AS with a baseline mpMRI. For PI-RADS 5 lesions, the positive predictive value (PPV) for upgrading at 3 years was only 41% while a PI-RADS 1–2 lesion had an NPV of 85% [25]. In another cohort of 295 men on AS, 3% had no MRI-visible tumor at baseline and there was significantly higher progression-free survival at 60 months for this group compared to those with MRI-visible lesions (97% versus 76%) [26]. Overall, these studies suggest that perhaps confirmatory biopsies may be delayed in patients with negative baseline mpMRI on AS as this confers a favorable prognosis.

#### Ability of radiological progression on mpMRI to predict pathologic progression

**Fig. 3:**
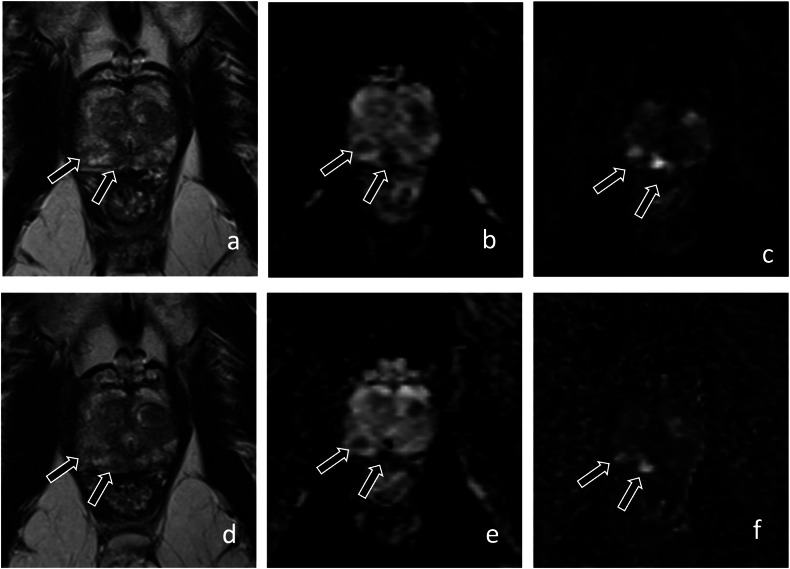
67-year-old man with PSA of 10.6 ng/mL and grade group 1 prostate cancer detected on systematic biopsy. Axial T2-weighted (**a**), apparent diffusion coefficient map (**b**), and high *b*-value (1400 s/mm^2^) diffusion-weighted image (**c**) show two PI-RADS 4 peripheral zone lesions on the right (arrows, **a**–**c**). A confirmatory biopsy revealed grade group 1 cancer on the lesions. The patient remained on active surveillance, and an MRI performed after 12 months revealed stable findings (arrows, **d**–**f**). (PRECISE score: 3-V) Repeat biopsy revealed grade group 2 cancer with small cribriform glands which was confirmed on radical prostatectomy

**Fig. 4:**
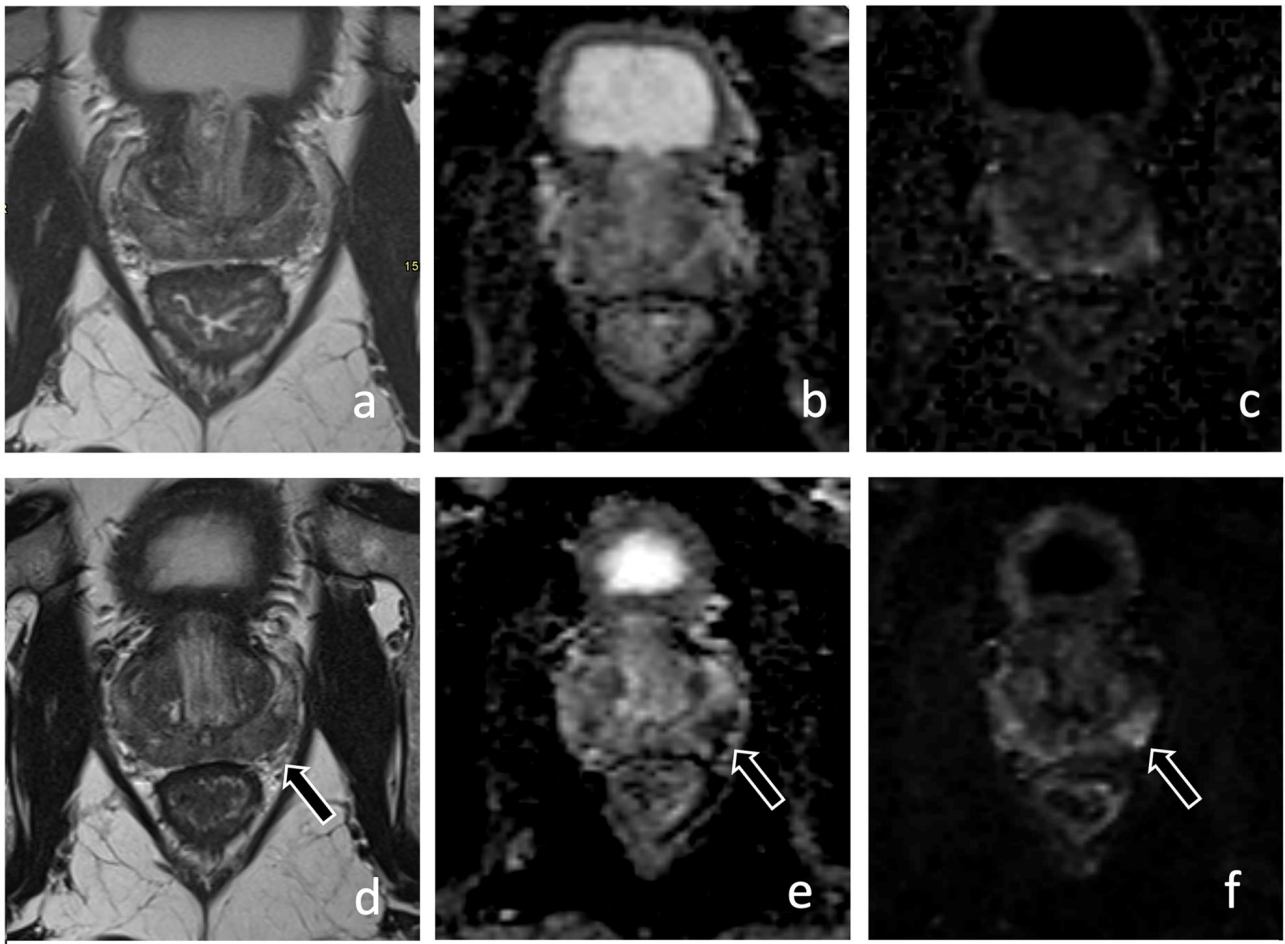
66-year-old man with PSA 4.3 ng/mL and grade group 1 prostate cancer on active surveillance. Axial T2-weighted (**a**), axial apparent diffusion coefficient map (**b**), and high *b*-value (1400 s/mm^2^) diffusion-weighted images (**c**) revealed mild diffuse changes in the peripheral zone (PI-RADS 2; white arrows, **a**–**c**). Follow-up MRI (**d**–**f**) performed at 24 months revealed persistent diffuse signal abnormalities in the peripheral zone, and a new 1.3 cm area of more intense restricted diffusion at the left base (PI-RADS 4, black arrow, **d**–**f**) (PRECISE score 4). MRI-guided biopsy revealed grade group 2 prostate cancer on the target

Changes in mpMRI results over time are difficult to interpret (Fig. 3). Chesnut et al studied 207 men on AS with baseline and 3-year prostate biopsies and mpMRI every 18 months. An increase in PI-RADS score had only a 41% PPV for reclassification at 3 years. Performing follow-up biopsy only for increased PI-RADS or change in clinical stage would avoid 681 biopsies per 1000 men but miss ≥ GG2 disease in 169 patients. Thus, performing biopsy only for radiological progression would miss an unacceptably high number of csPCa and follow-up biopsies regardless of mpMRI findings should still be standard [27]. Another study evaluated whether progression on mpMRI could predict pathological progression from GG1 to ≥ GG2 and found a PPV of MRI change for pathologic progression to csPCa of 69% with NPV 70% (mean interval between mpMR = 28.3 months) [28]. A retrospective study of 364 patients found that the PPV of progression on serial mpMRIs (at 12, 36, and 84 months after diagnosis; median follow-up time 36 months) ranged from 33–50% depending on what radiological progression criteria were used (size of the lesion, increase in PI-RADS, etc) for pathologic upgrading [29]. These results demonstrate that changes in serial mpMRIs may be difficult to understand and thus scheduled biopsies are still a critical part of AS protocols (Fig. 4); furthermore, mpMRI progression should only be used to trigger biopsy, not conversion to active treatment.

Luzzago et al tested the ability of serial mpMRI scans (recommended at 12, 36, and 84 months after diagnosis) to exclude progression to csPCa during AS and found that patients without radiological progression and with PSA density < 0.15 ng/mL could likely forego surveillance biopsies since < 5% of these patients experienced pathologic progression, highlighting the valuable additive benefit of serum biomarkers [29]. Another study examined 100 men on AS who underwent baseline and yearly mpMRI, did not undergo confirmatory biopsy, and had follow-up biopsies that were triggered by radiographic progression or patient or provider preference due to abnormal Digital rectal exam (DRE) and/or abnormal PSA density. The PPV, NPV, sensitivity, and specificity for detection of csPCa by surveillance mpMRI at 3 years were 45%, 89%, 61%, and 80% [30]. Similarly, a systematic review and meta-analysis of 2240 patients demonstrated pooled sensitivity and specificity of mpMRI progression to detect pathologic progression of 59% and 75%. Depending on the prevalence of PCa progression, the pooled PPV ranged from 37–50% and the pooled NPV ranged from 81–88%. Imaging intervals in this study were most commonly 12–24 months [31]. Overall, this suggests that MRI alone cannot rule out PCa progression and requires a combined approach with other biomarkers (PSA, PSAD, PHI) to determine surveillance timing [31, 32].

#### Stability of mpMRI lesions while on AS

Other studies have examined the stability of MRI lesions over time. Rais-Bahrami et al demonstrated the stability of small lesions (≤ 7 mm) detected on mpMRI and found that 86% were benign and 14% were GG1. These lesions demonstrated no significant change in size over a mean imaging period of 2.3 years [33]. This data suggests that for patients on AS with a small index lesion, a surveillance imaging interval of at least 2 years may be reasonable [33]. Another retrospective review of 364 patients studied the natural history of mpMRI lesions during AS and found a 27% probability of mpMRI progression at the first scan and lower rates of progression over time (2–4 scans) with a median follow-up of 36 months [29]. This data suggests that lengthening imaging intervals over time may be reasonable given the increased radiological stability of lesions over time. Longer imaging intervals may also be beneficial in settings with limited access to mpMRI and would lower cost.

### PRECISE score

The European School of Oncology introduced the “Prostate Cancer Radiological Estimation of Change in Sequential Evaluation” (PRECISE) score in 2016 to assess the likelihood of clinically significant radiological change based on a 5-point likelihood scale [34]. The PRECISE score of 1–2 represents resolution/improvement of previously suspicious MRI findings, a PRECISE score of 3 indicates stability, and a PRECISE score of 4–5 corresponds to progression. Studies have shown good interobserver reproducibility of the PRECISE score, both per patient and per scan level and that data were especially reproducible when grouped by absence versus presence of radiological progression (PRECISE 1–3 versus 4–5) [35].

Caglic et al examined 295 men on AS with a follow-up of 52 months. At a cut-off value of ≥ 4, PRECISE scoring had a sensitivity, specificity, PPV, and NPV for predicting pathologic progression of 76%, 89%, 52%, and 96%, respectively (AUC 0.82). The authors concluded that PRECISE scores of 1–3 have a high NPV which could limit the need for re-biopsy, while PRECISE 4–5 had a moderate PPV and should lead to closer surveillance or re-biopsy [26]. Furthermore, another study examined the ability of the PRECISE score to predict AS disqualification and found that no men with a PRECISE score of 1–2 were disqualified from AS over 4 years of follow-up [17]. Thus, the current literature suggests that men with low PRECISE scores may be able to defer follow-up biopsy or de-intensify surveillance.

Other studies have shown the inability of the PRECISE score to predict pathologic progression. Bhanji et al examined 163 men with mpMRI-informed diagnostic and confirmatory biopsies who had surveillance biopsies within 6 months of an interval mpMRI and found no statistically significant increase in the rate of pathologic progression with increased PRECISE score (PRECISE 1–2: 24%, PRECISE 3: 23%, PRECISE 4–5: 38%, *p* = 0.11). Conversely, the authors found a significant increase in the rate of pathologic progression with increasing PI-RADS score [36]. Another study examining 111 men with 1 year of surveillance mpMRI with biopsy showed more upgrading in men with a PRECISE score of 4–5 (41%) compared to men with a PRECISE score of 1–3 (30%). However, the high rate of upgrading in men without radiological progression indicates that systematic and mpMRI-targeted biopsy should still be performed at 1 year in all patients with positive mpMRI, regardless of PRECISE score. On the other hand, they observed only 10% upgrading in men with negative mpMRI and PRECISE score 3, suggesting that confirmatory biopsy at 1 year could potentially be deferred after discussion of the risks/benefits of biopsy [37].

**Fig. 5:**
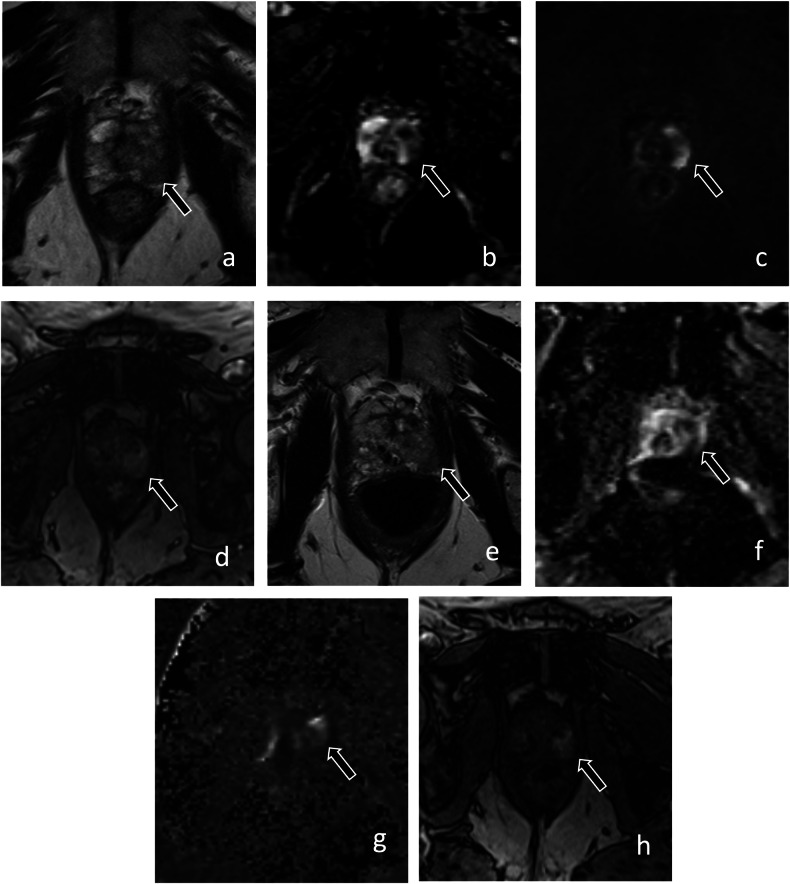
77-year-old man with PSA of 6 ng/mL. Axial T2-weighted (**a**), apparent diffusion coefficient (ADC) map (**b**), high *b*-value (1400 s/mm^2^) diffusion-weighted (**c**), and T1-weighted dynamic contrast-enhanced images revealed a 1.4 cm left apical peripheral zone PI-RADS 4 lesion (arrows, **a**–**d**). The T2-weighted images had some motion-related blurring. MRI-guided biopsy revealed grade group 2 cancer with 10% pattern 4. The patient opted for active surveillance. The lesion could be seen on follow-up MRI (arrows, **e**–**h**), but the assessment of the ADC map and diffusion-weighted images was limited by geometric distortion from rectal gas (PRECISE score: X)

**Fig. 6:**
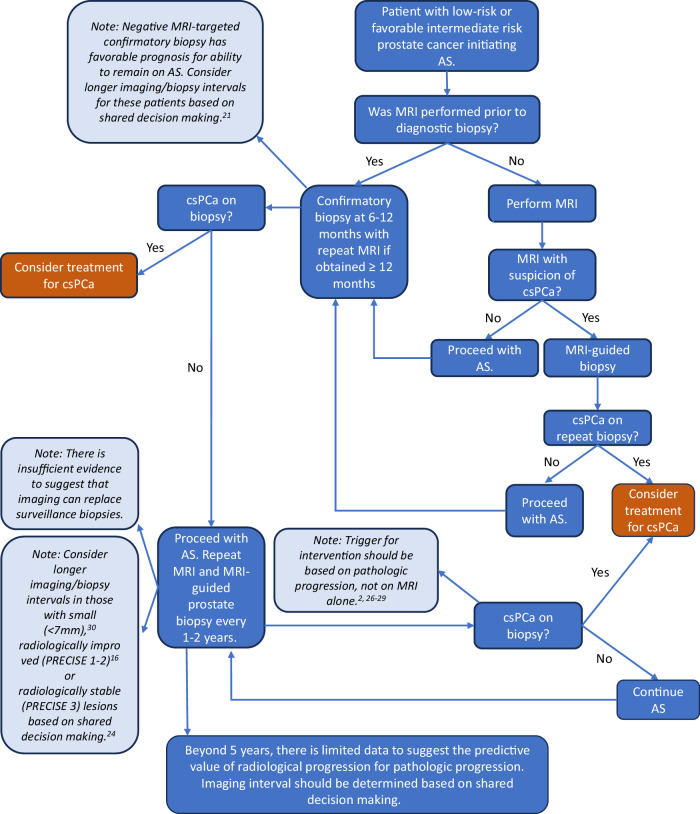
Proposed algorithm for the use of multiparametric MRI during active surveillance. AS, active surveillance; csPCa, clinically significant prostate cancer; MRI, magnetic resonance imaging; PRECISE, prostate cancer radiological estimation of change in sequential evaluation

Given the small number of publications from the US on this subject, it is reasonable to infer that the implementation of PRECISE in clinical settings within the US may be limited. Concerns about the inter-reader agreement and variability in image quality that can compromise the ability to accurately detect changes on serial exams probably constitute a barrier to the adoption of the system (Fig. 5) [38]. The introduction of a category “X” for exams with insufficient image quality in the recently revised PRECISE recommendations (PRECISE version 2) underscores the importance of high-quality images for an accurate assessment of MR findings in this setting [39]. Ongoing efforts to improve prostate MR image quality, such as the American College of Radiology Learning Network, and further refinements of the system that are still needed after the recent update, particularly with respect to setting specific quantitative thresholds for determining a significant increase in size and/or conspicuity, may help increase the system’s performance and its acceptance in practice [39–42]. Another noticeable change in PRECISE version 2, was the division of score 3 (stable MRI) into 3-V and 3-NonV for patients with MRI-visible (i.e., PI-RADS ≥ 3) and MRI-invisible disease, respectively, to indicate these two groups of patients may have different outcomes. A proposed algorithm for the use of mpMRI and PRECISE score during AS, based on the data presented above, is shown in Fig. 6.

### Other imaging techniques (MicroUS, PSMA PET)

#### MicroUS

Microultrasound (microUS) is a high-resolution imaging modality for transrectal ultrasonography that could potentially replace mpMRI for follow-up imaging of patients on AS. Many US healthcare systems currently have difficulty meeting demands for prostate MRI which is being increasingly ordered for screening, monitoring, and staging. Studies have shown both a benefit of microUS over mpMRI for the detection of csPCa and a benefit for simultaneous use of both modalities [43]. Specifically for AS, one study compared MicroUS-targeted biopsy to mpMRI-targeted biopsy (both with systematic biopsy) for 1-year confirmatory biopsy and showed a sensitivity of 94% and 100% and NPV of 89% and 100%, respectively, for detection of csPCa [44]. Thus, microUS demonstrated a similar ability to detect csPCa compared to mpMRI in the confirmatory biopsy setting. Further research is needed in this area before formal recommendations can be made.

#### Prostate membrane-specific antigen positron emission tomography (PSMA PET)

PET using PSMA-radioligands is being increasingly used in the initial staging of intermediate unfavorable and high-risk PCa and in the setting of suspected recurrence [45, 46]. Few studies exist regarding the utility of PSMA PET for monitoring patients on AS. The prospective PASPoRT study examined 141 patients on AS who all had baseline mpMRI-informed biopsy and underwent PSMA PET followed by targeted biopsy. A PSMA-targeted biopsy was required in 32% of patients and 9% experienced pathologic upgrading. Upgrading was most commonly observed in patients with higher PSA density and negative mpMRI. Overall, the number needed to scan (NNS) was 11; however, the NNS in the subset of patients with PSA density ≥ 0.15 and negative mpMRI was only 2.8 [47]. The PRIMARY study examined men with suspected PCa who underwent mpMRI and PSMA PET followed by a systematic and targeted biopsy. Combined PSMA plus mpMRI significantly improved NPV (91% versus 72%) and sensitivity (97% versus 83%) compared to mpMRI alone, suggesting that biopsy could potentially be avoided or deferred in men with negative combined imaging [48]. Other studies have demonstrated the utility of PSMA PET/computed tomography in detecting MRI-invisible lesions for patients on AS, thus improving risk stratification and detection of csPCa [49, 50]. Although there may be a role for PSMA PET in some patients with AS, further research is clearly needed and there are ongoing trials addressing this [51, 52].

## Conclusions

The US guidelines provide guidance about the use of mpMRI in diagnostic and confirmatory biopsy settings. However, no definite recommendations are made about imaging intervals for men on AS. The current literature shows promise regarding ways to minimize the burden of AS and reduce the risk of misclassification, however further work is needed to more clearly define the role of mpMRI in AS in terms of imaging intervals and when a biopsy can be deferred based on imaging findings.

The lack of evidence about the utilization and timing of mpMRI in AS can potentially lead to over or underutilization of this relatively scarce resource. Furthermore, defining the optimal imaging interval for mpMRI could improve cost-effectiveness by eliminating unnecessarily frequent mpMRIs for men on AS. There is also ongoing interest in the use of biomarkers for men on AS to be used in conjunction with mpMRI, although data to support their use is currently lacking.

Future research should address some of these important issues, allowing the US (and also European) guidelines to provide more concrete recommendations about imaging intervals and utilization of mpMRI of the prostate during AS.

## Abbreviations

AS: Active surveillance
AUA: American Urological Association
GG: Grade group
microUS: Microultrasound
mpMRI: Multiparametric magnetic resonance imaging
NCCN: National Comprehensive Cancer Network
NPV: Negative predictive value
PCa: Prostate cancer
PET: Positron emission tomography
PI-RADS: Prostate Imaging Reporting and Data System
PSA: Prostate-specific antigen
PPV: Positive predictive value
PSMA: Prostate membrane-specific antigen
US: United States

## References

[1] Choo R, Klotz L, Danjoux C et al (2002) Feasibility study: watchful waiting for localized low to intermediate grade prostate carcinoma with selective delayed intervention based on prostate specific antigen, histological and/or clinical progression. J Urol 167:1664–1669 11912384

[2] Carter HB, Walsh PC, Landis P, Epstein JI (2002) Expectant management of nonpalpable prostate cancer with curative intent: preliminary results. J Urol 167:1231–1234 11832703

[3] Tosoian JJ, Mamawala M, Epstein JI et al (2015) Intermediate and longer-term outcomes from a prospective active-surveillance program for favorable-risk prostate cancer. J Clin Oncol 33:3379–3385. 10.1200/JCO.2015.62.5764 26324359 10.1200/JCO.2015.62.5764 PMC4863946

[4] Klotz L, Vesprini D, Sethukavalan P et al (2015) Long-term follow-up of a large active surveillance cohort of patients with prostate cancer. J Clin Oncol 33:272–277. 10.1200/JCO.2014.55.1192 25512465 10.1200/JCO.2014.55.1192

[5] Cooperberg MR, Meeks W, Fang R et al (2023) Time trends and variation in the use of active surveillance for management of low-risk prostate cancer in the US. JAMA Netw Open 6:E231439. 10.1001/JAMANETWORKOPEN.2023.1439 36862409 10.1001/jamanetworkopen.2023.1439 PMC9982696

[6] Liu Y, Hall IJ, Filson C, Howard DH (2021) Trends in the use of active surveillance and treatments in Medicare beneficiaries diagnosed with localized prostate cancer. Urol Oncol 39:432.e1–432.e10. 10.1016/J.UROLONC.2020.11.024 33308973 10.1016/j.urolonc.2020.11.024 PMC8374746

[7] Loeb S, Byrne NK, Wang B et al (2020) Exploring variation in the use of conservative management for low-risk prostate cancer in the veterans affairs healthcare system. Eur Urol 77:683–686. 10.1016/J.EURURO.2020.02.004 32098730 10.1016/j.eururo.2020.02.004 PMC7250727

[8] Agrawal V, Ma X, Hu JC et al (2021) Active surveillance for men with intermediate risk prostate cancer. J Urol 205:115–121. 10.1097/JU.0000000000001241 32658588 10.1097/JU.0000000000001241

[9] Leapman MS, Wang R, Loeb S et al (2023) Use of monitoring tests among patients with localized prostate cancer managed with observation. J Urol 209:710–718. 10.1097/JU.000000000000315936753746 10.1097/JU.0000000000003159

[10] Turkbey B, Rosenkrantz AB, Haider MA et al (2019) Prostate Imaging Reporting and Data System Version 2.1: 2019 Update of Prostate Imaging Reporting and Data System Version 2. Eur Urol 76:340–351. 10.1016/J.EURURO.2019.02.033 30898406 10.1016/j.eururo.2019.02.033

[11] Sanda MG, Cadeddu JA, Kirkby E et al (2018) Clinically localized prostate cancer: AUA/ASTRO/SUO guideline. Part I: risk stratification, shared decision making, and care options. J Urol 199:683–690. 10.1016/J.JURO.2017.11.095 29203269 10.1016/j.juro.2017.11.095

[12] Sanda MG, Cadeddu JA, Kirkby E et al (2018) Clinically localized prostate cancer: AUA/ASTRO/SUO guideline. Part II: recommended approaches and details of specific care options. J Urol 199:990–997. 10.1016/J.JURO.2018.01.002 29331546 10.1016/j.juro.2018.01.002

[13] Schaeffer EM, Srinivas S, Adra N et al (2022) NCCN Guidelines® Insights: Prostate Cancer, Version 1.2023. J Natl Compr Canc Netw 20:1288–1298. 10.6004/JNCCN.2022.0063 36509074 10.6004/jnccn.2022.0063

[14] Mottet N, van den Bergh RCN, Briers E et al (2021) EAU-EANM-ESTRO-ESUR-SIOG Guidelines on Prostate Cancer-2020 Update. Part 1: screening, diagnosis, and local treatment with curative intent. Eur Urol 79:243–262. 10.1016/J.EURURO.2020.09.042 33172724 10.1016/j.eururo.2020.09.042

[15] Mottet N, Cornford P, van den Bergh RCN et al EAU-EANM-ESTRO-ESUR-SIOG Guidelines on Prostate Cancer-2023 Update. uroweb.org/guidelines/prostate-cancer/summary-of-changes. Accessed 24 Mar 2024

[16] Lam TBL, MacLennan S, Willemse PPM et al (2019) EAU-EANM-ESTRO-ESUR-SIOG Prostate Cancer Guideline Panel Consensus Statements for Deferred Treatment with Curative Intent for Localised Prostate Cancer from an International Collaborative Study (DETECTIVE Study). Eur Urol 76:790–813. 10.1016/J.EURURO.2019.09.020 31587989 10.1016/j.eururo.2019.09.020

[17] Dieffenbacher S, Nyarangi-Dix J, Giganti F et al (2021) Standardized magnetic resonance imaging reporting using the prostate cancer radiological estimation of change in sequential evaluation criteria and magnetic resonance imaging/transrectal ultrasound fusion with transperineal saturation biopsy to select men on active surveillance. Eur Urol Focus 7:102–110. 10.1016/J.EUF.2019.03.001 30878348 10.1016/j.euf.2019.03.001

[18] Schiavina R, Droghetti M, Novara G et al (2021) The role of multiparametric MRI in active surveillance for low-risk prostate cancer: the ROMAS randomized controlled trial. Urol Oncol 39:433.e1–433.e7. 10.1016/J.UROLONC.2020.10.018 33191117 10.1016/j.urolonc.2020.10.018

[19] Klotz L, Pond G, Loblaw A et al (2020) Randomized study of systematic biopsy versus magnetic resonance imaging and targeted and systematic biopsy in men on active surveillance (ASIST): 2-year postbiopsy follow-up. Eur Urol 77:311–317. 10.1016/J.EURURO.2019.10.007 31708295 10.1016/j.eururo.2019.10.007

[20] Shapiro DD, Gregg JR, Lim AH et al (2021) Comparing confirmatory biopsy outcomes between MRI-targeted biopsy and standard systematic biopsy among men being enrolled in prostate cancer active surveillance. BJU Int 127:340–348. 10.1111/BJU.15100 32357283 10.1111/bju.15100 PMC9798524

[21] Schoots IG, Nieboer D, Giganti F et al (2018) Is magnetic resonance imaging-targeted biopsy a useful addition to systematic confirmatory biopsy in men on active surveillance for low-risk prostate cancer? A systematic review and meta-analysis. BJU Int 122:946–958. 10.1111/BJU.14358 29679430 10.1111/bju.14358

[22] Ma TM, Tosoian JJ, Schaeffer EM et al (2017) The role of multiparametric magnetic resonance imaging/ultrasound fusion biopsy in active surveillance. Eur Urol 71:174–180. 10.1016/J.EURURO.2016.05.021 27236496 10.1016/j.eururo.2016.05.021

[23] Bloom JB, Hale GR, Gold SA et al (2019) Predicting Gleason group progression for men on prostate cancer active surveillance: role of a negative confirmatory magnetic resonance imaging-ultrasound fusion biopsy. J Urol 201:84–90. 10.1016/J.JURO.2018.07.051 30577395 10.1016/j.juro.2018.07.051 PMC8274955

[24] Yerram NK, Long L, O’Connor LP et al (2021) Magnetic resonance imaging-targeted and systematic biopsy for detection of grade progression in patients on active surveillance for prostate cancer. J Urol 205:1352–1360. 10.1097/JU.0000000000001547 33356479 10.1097/JU.0000000000001547 PMC9677228

[25] Kornberg Z, Cowan JE, Westphalen AC et al (2019) Genomic prostate score, PI-RADS^TM^ version 2 and progression in men with prostate cancer on active surveillance. J Urol 201:300–306. 10.1016/J.JURO.2018.08.047 30179620 10.1016/j.juro.2018.08.047

[26] Caglic I, Sushentsev N, Gnanapragasam VJ et al (2021) MRI-derived PRECISE scores for predicting pathologically-confirmed radiological progression in prostate cancer patients on active surveillance. Eur Radiol 31:2696–2705. 10.1007/S00330-020-07336-0 33196886 10.1007/s00330-020-07336-0 PMC8043947

[27] Chesnut GT, Vertosick EA, Benfante N et al (2020) Role of changes in magnetic resonance imaging or clinical stage in evaluation of disease progression for men with prostate cancer on active surveillance. Eur Urol 77:501–507. 10.1016/J.EURURO.2019.12.009 31874726 10.1016/j.eururo.2019.12.009 PMC7096768

[28] Felker ER, Wu J, Natarajan S et al (2016) Serial magnetic resonance imaging in active surveillance of prostate cancer: incremental value. J Urol 195:1421–1427. 10.1016/J.JURO.2015.11.055 26674305 10.1016/j.juro.2015.11.055 PMC4900685

[29] Luzzago S, Piccinelli ML, Mistretta FA et al (2022) Repeat MRI during active surveillance: natural history of prostatic lesions and upgrading rates. BJU Int 129:524–533. 10.1111/BJU.15623 34687137 10.1111/bju.15623

[30] Amin A, Scheltema MJ, Shnier R et al (2020) The magnetic resonance imaging in active surveillance (MRIAS) trial: use of baseline multiparametric magnetic resonance imaging and saturation biopsy to reduce the frequency of surveillance prostate biopsies. J Urol 203:910–917. 10.1097/JU.0000000000000693 31825297 10.1097/JU.0000000000000693

[31] Rajwa P, Pradere B, Quhal F et al (2021) Reliability of serial prostate magnetic resonance imaging to detect prostate cancer progression during active surveillance: a systematic review and meta-analysis. Eur Urol 80:549–563. 10.1016/J.EURURO.2021.05.001 34020828 10.1016/j.eururo.2021.05.001

[32] Schwen ZR, Mamawala M, Tosoian JJ et al (2020) Prostate Health Index and multiparametric magnetic resonance imaging to predict prostate cancer grade reclassification in active surveillance. BJU Int 126:373–378. 10.1111/BJU.15101 32367635 10.1111/bju.15101

[33] Rais-Bahrami S, Türkbey B, Rastinehad AR et al. (2014) Natural history of small index lesions suspicious for prostate cancer on multiparametric MRI: recommendations for interval imaging follow-up. Diagn Interv Radiol 20:293–298. 10.5152/DIR.2014.13319 24808435 10.5152/dir.2014.13319 PMC4463272

[34] Moore CM, Giganti F, Albertsen P et al (2017) Reporting magnetic resonance imaging in men on active surveillance for prostate cancer: the PRECISE recommendations-a report of a European School of Oncology Task Force. Eur Urol 71:648–655. 10.1016/J.EURURO.2016.06.011 27349615 10.1016/j.eururo.2016.06.011

[35] Giganti F, Pecoraro M, Stavrinides V et al (2020) Interobserver reproducibility of the PRECISE scoring system for prostate MRI on active surveillance: results from a two-centre pilot study. Eur Radiol 30:2082–2090. 10.1007/S00330-019-06557-2 31844959 10.1007/s00330-019-06557-2 PMC7062656

[36] Bhanji Y, Mamawala M, de la Calle CM et al (2023) Prostate cancer radiological estimation of change in sequential evaluation (PRECISE) magnetic resonance imaging scoring to predict clinical outcomes in active surveillance for grade group 1 prostate cancer. Urology 180. 10.1016/J.UROLOGY.2023.07.019 10.1016/j.urology.2023.07.019 37536582

[37] Osses DF, Drost FJH, Verbeek JFM et al (2020) Prostate cancer upgrading with serial prostate magnetic resonance imaging and repeat biopsy in men on active surveillance: are confirmatory biopsies still necessary? BJU Int 126:124–132. 10.1111/BJU.15065 32232921 10.1111/bju.15065 PMC7383866

[38] Giganti F, Ng A, Asif A et al (2023) Global variation in magnetic resonance imaging quality of the prostate. Radiology 309. 10.1148/RADIOL.231130 10.1148/radiol.231130 37815448

[39] Englman C, Maffei D, Allen C et al (2024) PRECISE version 2: updated recommendations for reporting prostate magnetic resonance imaging in patients on active surveillance for prostate cancer. Eur Urol. 10.1016/J.EURURO.2024.03.014 10.1016/j.eururo.2024.03.014 38556436

[40] Purysko AS, Tempany C, Macura KJ et al (2023) American College of Radiology initiatives on prostate magnetic resonance imaging quality. Eur J Radiol 165. 10.1016/J.EJRAD.2023.110937 10.1016/j.ejrad.2023.110937 PMC10461171 37352683

[41] Sanmugalingam N, Sushentsev N, Lee KL et al (2023) The PRECISE recommendations for prostate MRI in patients on active surveillance for prostate cancer: a critical review. AJR Am J Roentgenol 221:649–660. 10.2214/AJR.23.29518 37341180 10.2214/AJR.23.29518

[42] Stavrinides V, Giganti F, Trock B et al (2020) Five-year outcomes of magnetic resonance imaging-based active surveillance for prostate cancer: a large cohort study. Eur Urol 78:443–451. 10.1016/J.EURURO.2020.03.035 32360049 10.1016/j.eururo.2020.03.035 PMC7443696

[43] Dias AB, Ghai S (2024) Prostate cancer diagnosis with micro-ultrasound: what we know now and new horizons. Radiol Clin North Am 62:189–197. 10.1016/J.RCL.2023.06.014 37973243 10.1016/j.rcl.2023.06.014

[44] Maffei D, Fasulo V, Avolio PP et al (2023) Diagnostic performance of microUltrasound at MRI-guided confirmatory biopsy in patients under active surveillance for low-risk prostate cancer. Prostate 83:886–895. 10.1002/PROS.24532 36960788 10.1002/pros.24532

[45] Fanti S, Goffin K, Hadaschik BA et al (2021) Consensus statements on PSMA PET/CT response assessment criteria in prostate cancer. Eur J Nucl Med Mol Imaging 48:469–476. 10.1007/S00259-020-04934-4 32617640 10.1007/s00259-020-04934-4 PMC7835167

[46] Seifert R, Emmett L, Rowe SP et al (2023) Second version of the prostate cancer molecular imaging standardized evaluation framework including response evaluation for clinical trials (PROMISE V2). Eur Urol 83:405–412. 10.1016/J.EURURO.2023.02.002 36935345 10.1016/j.eururo.2023.02.002

[47] Heetman JG, Lavalaye J, Polm PD et al (2023) Gallium-68 prostate-specific membrane antigen positron emission tomography/computed tomography in active surveillance for prostate cancer trial (PASPoRT). Eur Urol Oncol. 10.1016/J.EUO.2023.05.004 10.1016/j.euo.2023.05.004 37296065

[48] Emmett L, Buteau J, Papa N et al (2021) The additive diagnostic value of prostate-specific membrane antigen positron emission tomography computed tomography to multiparametric magnetic resonance imaging triage in the diagnosis of prostate cancer (PRIMARY): a prospective multicentre study. Eur Urol 80:682–689. 10.1016/J.EURURO.2021.08.002 34465492 10.1016/j.eururo.2021.08.002

[49] Liu J, Santucci J, Woon DTS et al (2024) A systematic review on prostate-specific membrane antigen positron emission tomography (PSMA PET) evaluating localized low- to intermediate-risk prostate cancer: a tool to improve risk stratification for active surveillance? Life (Basel) 14:76. 10.3390/LIFE14010076 38255691 10.3390/life14010076 PMC10817570

[50] Akcay K, Kibar A, Sahin OE et al (2023) Prediction of clinically significant prostate cancer by [68 Ga]Ga-PSMA-11 PET/CT: a potential tool for selecting patients for active surveillance. Eur J Nucl Med Mol Imaging. 10.1007/S00259-023-06556-Y 10.1007/s00259-023-06556-y 38112777

[51] Bagguley D, Harewood L, McKenzie D et al (2023) The CONFIRM trial protocol: the utility of prostate-specific membrane antigen positron emission tomography/computed tomography in active surveillance for prostate cancer. BJU Int 4:27–36. 10.1111/BJU.16214 10.1111/bju.16214 37904302

[52] Gondoputro W, Doan P, Katelaris A et al (2023) 68Ga-PSMA-PET/CT in addition to mpMRI in men undergoing biopsy during active surveillance for low- to intermediate-risk prostate cancer: study protocol for a prospective cross-sectional study. Transl Androl Urol 12:1598–1606. 10.21037/TAU-22-708/COIF 10.21037/tau-22-708 PMC10643393 37969779

